# Professional Quality of Life of Foster and Kinship Carers in Australia, United Kingdom, and the United States: A Scoping Review

**DOI:** 10.1177/15248380231213322

**Published:** 2023-12-01

**Authors:** Helen McLaren, Emi Patmisari, Yunong Huang

**Affiliations:** 1 Flinders University, Adelaide, SA, Australia

**Keywords:** foster care, kinship care, quality of life, secondary traumatic stress, burnout, compassion satisfaction

## Abstract

Professional quality of life (ProQOL) refers to workers’ subjective feelings associated with work involved in helping others who have experienced trauma. It consists of positive and negative aspects, that is, subscales of compassion satisfaction, and burnout and secondary traumatic stress. Foster and kinship caring inherently involves risks associated with exposure to the trauma responses of children in their care. This exposure can lead to poor ProQOL, carer attrition, and placement instability. While limited studies specifically explore ProQOL of carers, many studies have examined factors and interventions related to ProQOL. However, there is a lack of synthesis of these studies. To fill such a research gap, we undertook a scoping review of 70 empirical studies from Australia, the United Kingdom, and the United States, published from 2012 to 2022 reporting on ProQOL, and its related factors and concepts. We applied a multilevel ecosocial construct to examine complex interrelationships between private and governance settings to better understand factors related to ProQOL of carers and interventions aimed to improve it in these dynamic systems. In our review, some studies showed positive outcomes for carers, such as reduced stress or burnout associated with training. However, there was insufficient attention to factors associated with ProQOL at relational and sociopolitical levels. It is crucial to improve carers’ ProQOL or well-being to ensure their retention and placement stability. Long-term systemic improvements require interventions across different levels of the system.

## Introduction

In Australia, more than 178,000 children and young people were unable to live safely at home in 2020 to 2021, with just over a quarter of them living in out-of-home care (Australian Institute of Health and Welfare, 2022), and the number continues to rise. The United Kingdom and United States also experience high and escalating rates of children in the care of the State (Corliss et al., 2022; Roehrkasse, 2021). Internationally, researchers show that children and young people in State care have poor social-emotional functioning (Jacobsen et al., 2020), physical and psychological functioning (Dubois-Comtois et al., 2021; Engler et al., 2022; Jacobsen et al., 2020), mental health problems (Haselgruber et al., 2020; Mancinelli et al., 2021), emotional and behavioral difficulties (Barboza et al., 2017; Paine et al., 2020), inferior academic outcomes (Nadorff et al., 2021; Washington et al., 2021), and complex needs associated with trauma (Engler et al., 2022; Kothari et al., 2020). Traumatic histories and associated behavioral difficulties of these children and young people can place significant stress on foster and kinship carers (Doley et al., 2015; Gleeson et al., 2016; Vanderfaeillie, Gypen et al., 2020; Vanderfaeillie, Van Den Abbeele et al., 2020), leading to higher levels of secondary traumatic stress and burnout than experienced by biological parents (Bergsund et al., 2020). Developing effective interventions that alleviate these negative experiences for carers is crucial to reducing carer attrition and strengthening both the care environment and placement stability. Research suggested that placement stability is positively associated with improved well-being, health outcomes, and life chances among children and young people in care (Dubois-Comtois et al., 2021; Mabille et al., 2022; Rubin et al., 2007).

Many researchers have sought to examine the factors associated with stress and well-being among foster and kinship carers (e.g., Doley et al., 2015; Gleeson et al., 2016) and the interventions aimed to reduce these feelings (e.g., Hutchings & Bywater, 2013; Midgley et al., 2019, 2021; Ranzato et al., 2021). A variety of factors have been found to associated with stress and well-being among carers, such as fostered children’s emotional and behavioral issues (Fergeus et al., 2019a, 2019b; Harnett et al., 2014; McKeough et al., 2017), the authoritative and reductionist attitudes of welfare workers (Fergeus et al., 2019b), and dealing with complex sociopolitical issues (Blythe et al., 2013). Many interventions have also been developed to reduce carers’ stress, such as the Incredible Years (IY) Parenting Program (Hutchings & Bywater, 2013); the Circle Program (CP) (Frederico et al., 2014), and attachment-centered parenting (Begum et al., 2020). However, there is a lack of research to integrate different factors about stress and well-being among foster and kinship carers and interventions to reduce these feelings in a systematic way. To the best of our knowledge, no previous publication has employed the methodology of a scoping review. To fill the research gap, this review provides a structured overview of empirical research on the factors associated with professional quality of life (ProQOL) and its related concepts and the interventions to improve these among foster and kinship carers. We focus on ProQOL because it is a collective term comprising both compassion satisfaction and compassion fatigue experienced by those who care for others exposed to traumatic stressors (Stamm, 2010), including carers of the children and young people in State care. Compassion fatigue includes two parts: burnout and secondary trauma (Stamm, 2010). ProQOL incorporates both positive and negative elements of care experiences and thus provides practitioners and researchers with a comprehensive indication of carers’ well-being and quality of life (Stamm, 2010). Understanding ProQOL is essential to improve the work experience for carers and the environment for the children in their care.

The aim of this research is not to provide a full systematic review, but to establish a contemporary foundation for future research. A scoping review which typically aims to identify the types of available evidence in a given field, identify key characteristics or factors related to a concept, and identify and analyze knowledge gaps (Munn et al., 2018) is therefore employed. We applied Wendt’s (2021) multilevel ecosocial approach to examine the interrelationship between private spheres and societal and governmental processes and carer ProQOL in the context of the interrelated dynamic systems. We limited the review to relevant literature from Australia, United Kingdom, and United States on the determinants of ProQOL and its related concepts as well as the interventions aimed to improve these for foster and kinship carers from 2012 to 2022. We focused on the three countries due to the Australian authors seeking evidence from the United Kingdom and United States in trend with Australia adopting child protection innovations from these two countries. Our research questions: (a) What are the factors associated with ProQOL or related concepts among foster and kinship carers as reported in research undertaken in Australia, the United Kingdom, and the United States? And (b) What are the models of intervention associated with the improvement of ProQOL or its related concepts for foster and kinship carers as reported in research undertaken in Australia, the United Kingdom, and the United States?

## Methods

Our approach followed the methodological framework developed by Arksey and O’Malley (2005) and subsequent methodological advancements by Levac et al. (2010) and Joanna Briggs Institute (Peters et al., 2021). Reviewing involved an iterative six-step technique: (a) establishing the research question; (b) defining the scope and scoping process; (c) locating relevant studies; (d) extracting data; (e) synthesizing reported results; and (f) unearthing implications for practice.

### Search Technique

We used the Population, Concept, and Context framework (Peters et al., 2021) to formulate the inclusion criteria. When our pilot search for “carer” AND “out-of-home care” AND “quality of life” synonyms returned relatively few items, and only from the United Kingdom. We expanded the inclusion criterion to include stress, well-being, satisfaction, negative affect, positive affect, and health-related quality of life as ProQOL subscale-related concepts as shown in Table 1.

**Table 1.** Framing the Scope and Question for the Review.

Population (P)	Concept (C)	Context (C)
Out-of-home carers, including foster carers, kinship carers, and foster parents	Professional quality of life, quality of life, compassion satisfaction, burnout, compassion fatigue, secondary traumatic stress, stress, well-being, satisfaction, positive affect, and negative affect	Original research conducted in Australia, United Kingdom, and United States, reported in English in peer reviewed journals

**Figure 1.**
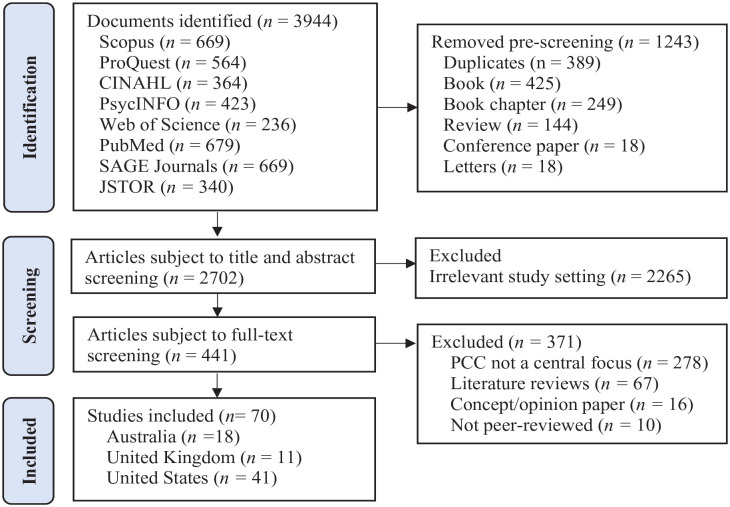
PRISMA (Preferred Reporting Items for Systematic reviews and Meta-Analyses) flow diagram of studies included.

Eight electronic databases were searched: Scopus, ProQuest, CINAHL, PsycINFO, Web of Science, PubMed, SAGE Journals, and JSTOR (Supplemental Material 1: Search strategy for each database) by the second author. A total of 3,944 references were identified for screening. Following the removal of duplicates and nonempirical journal articles, 2,702 studies were exported to Covidence (https://app.covidence.org), a tool for online inclusion/exclusion screening, for further screening. A two-step process involving screening of title and abstracts first and then full-text review was performed independently by the first and second authors, In each step, disagreement was discussed until a consensus was achieved. A total of 70 studies were included in final analysis. Figure 1 shows the study selection flowchart of this review.

### Studies Included

Articles for inclusion (*n* *=* 70) consisted of 18 Australian studies of determinants of ProQOL or its related concepts (*n* *=* 15) and measurement of ProQOL or its related concepts associated with interventions (*n* *=* 3), 11 studies from the United Kingdom (determinants *n* *=* 5; interventions *n* *=* 6) and 41 from the United States (determinants *n* *=* 25; interventions *n* *=* 16). Studies were divided by country and type (determinants or interventions).

### Data Synthesis

A spreadsheet was created to chart the data that answers the research questions. Information on authorship, aim of research, methods of research, and key findings were recorded and synthesized in summary format. The process was carried out by the second author and discussed with other authors. Disagreement was discussed until a consensus was achieved. Code and theme development was guided by ecosocial model to emphasize relationships between human condition and the settings in which individual and collective action takes place (Wendt, 2021). The ecosocial model appreciated the complex interplay between multifaceted conditions affecting ProQOL or its related concepts and interventions associated with the caring role. It consists of three nested layers: the individual which focuses on personal action frameworks like carer’s interpersonal relationships, roles, and activities; the relational which addresses interconnections and links between ProQOL or its related concepts and individuals’ immediate settings including funding agencies, service providers, and foster care agencies and serves as a platform for stakeholders to work together; and the sociopolitical which centers on political decision-making and assists in identifying connections between ProQOL or its related concepts and interventions and policy, structures, and bureaucracy such as foster care system (Wendt, 2021). The model provided a comprehensive framework to organize different factors associated with ProQOL and its related concepts as well as the interventions to improve them in a systematic way, enabling observation of the system influences across determinant- and intervention-related study foci across the three countries. In this review, we categorized studies as individual focused if they addressed the factors or interventions centered on the carers’ personal or social life and circumstances. Studies were categorized as relational focused if they examined the factors or interventions related to funding agencies, service providers, and foster care agencies and addressed the interactions between carers, child welfare organizations, and other groups working together to ensure the welfare and development of the children and young people. Studies were regarded as sociopolitical focused if they focused on the factors and interventions related to foster care systems or policies.

## Results

This section describes the characteristics of the 70 studies. They consisted of 18 Australian studies (quantitative *n* *=* 8, qualitative *n* *=* 6, and mixed methods *n* *=* 4), 11 UK studies (quantitative *n* *=* 4, qualitative *n* *=* 3, and mixed methods *n* *=* 4), and 41 US studies (quantitative *n* *=* 30, qualitative *n* *=* 7, and mixed methods *n* *=* 4). Most studies were not discretely focused on ProQOL; however, 15 Australian studies had some attention toward determinants of ProQOL or its related concepts, and three reporting on measures of ProQOL or its related concepts associated with interventions. Of the UK studies, five reported determinants of ProQOL, its aspects, or its related concepts and six on interventions, compared to 25 US studies reporting determinants of ProQOL or its related concepts and 16 on interventions (Table 2).

**Table 2.** Summary of Studies Included.

Australia—Studies Including Professional Quality of Life (ProQOL) or Its Related Concepts			
Authors	Aim	Methods	Relevant Results
Blythe et al. (2013)	To explore challenges experienced by women who are long-term foster carers.	Qualitative study of carers (*n* *=* 20), narrative storytelling interviews.	Carer stress associated with scrutiny or criticism by child protection workers. Carer frustration associated with workers’ broken promises, interference, and disempowering carers in decision making.
Blythe et al. (2012)	To examine stigma experienced by women who are long-term foster carers.	Qualitative study of carers (*n* *=* 20), interviews.	Carers stress, marginalization, disempowerment, and social isolation had associations with negative stereotypes of carers, such as “in it for the money” uncaring, and lacking commitment.
Breman et al. (2018)	To examine impact of child-perpetrated violence on kinship carers.	Mixed methods study of carers, online survey on violence types, frequency, and impact (*n* *=* 101), interviews (*n* *=* 22).	Carer stress, poor mental health and deterioration of physical health associated with daily experiences of violence and abuse by the children and other family members toward them.
Fergeus et al. (2019a)	To explain impact of unmet needs of foster and kinship carers.	Quantitative study of carers (*n* *=* 68), Carer and User Expectation of Services-Carer survey.	Carer stress, exhaustion, lethargy, poor sleep, no time for self-care, were associated with children’s physical, emotional and intellectual needs, and child-perpetrated aggression. Stress compounded by lack of historical information from child protection workers about children’s health, legal, and family/social contexts.
Fergeus et al. (2019b)	To explore experiences of foster and kinship carers with roles as mental health and early intervention advocates.	Qualitative study of carers (*n* *=* 31), either focus group or interviews.	Carer stress associated with perceived lack of systemic support, failed advocacy attempts to garner support for children’s mental health and behavioral problems, and authoritative and reductionist attitudes by child protection workers. Kinship carer stress was associated with feelings of having no support at all.
Fernandes et al. (2021)	To identify contexts of inequity and injustice as described by grandparent carers.	Qualitative study of carers (*n* *=* 69), either focus group or interviews.	Carers stress associated with demands of the child protection system, limited welfare supports and inaccessible service routes. Distress, marginalization, and disempowerment were compounded by inequity associated with being a grandparent carer.
Harding et al. (2020)	To compare the perceived well-being of foster and kinship carers.	Quantitative study of carers (*n* *=* 326), Brief Assessment Checklist for Children (BAC-C), Parent Mental Health Scale, Parent Stress Scale (PSS), scaled services and resources questions.	Perceived well-being was similar for both foster and kinship carers. Foster carers reported less stress and mental health issues than kinship carers. Kinship carers had less access to support, training, and services than foster carers.
Harnett et al. (2014)	To compare grandparent and foster carer characteristics, perception of child functioning, social support, and day-to-day difficulties, on carer stress.	Quantitative study of carers (*n* *=* 114) and children (*n* *=* 180), Strengths and Difficulties Questionnaire (SDQ) and Vineland Adaptive Behavior Scales, Parenting Stress Index (PSI), Child Abuse Potential Inventory (CA pi), Significant Other Scale, Daily Hassles Scale ratings.	Stress associated with child behavioral problems higher in grandparent carer compared to foster carers. Grandparents scored more daily hassles, despite the children showing better behavioral and adaptive functioning. Grandparents scored higher on the CAPI and the PSI scales. Grandparents had lower emotional and practical support, more financial difficulties, and more family-related stress generally.
Kiraly et al. (2020)	To explore experiences and support needs of kinship carers, mostly 18- to 30-year-old siblings and aunts.	Qualitative study of carers (*n* *=* 41), interviews.	Most stress associated with financial problems, that is, struggled to pay for children’s school uniforms and transportation costs. Stress also associated with parenting distressed children, juggling own studies and employment while caring, interfamilial conflicts, and lack of support.
Kiraly et al. (2015)	To explore views of kinship carers on supporting children’s connections to birth family, culture, and country.	Mixed methods study of carers (*n* *=* 430), focus groups (*n* *=* 10), and interviews (*n* *=* 73).	Carer stress associated with service unavailability when needed, poor understanding of Indigenous cultures by child protection workers, and poor communication on case planning, decision-making, and contact arrangements, and no support for carers own emotional needs.
McKeough et al. (2017)	To explain foster carer stress and well-being related to role-specific contexts, including challenging behaviors, birth family contact, training, and support.	Quantitative study of carers (*n* *=* 133), 10-item challenges questionnaire, Depression Anxiety Stress Scale, PSI, training supports questions.	Carers scored severe/extreme stress (13%) and mild-to-moderate stress (28%). Main source of stress was children’s challenging behaviors, followed by birth family contact. Added pressure associated with time constraints and role demands, including burden of face-to-face training.
McPherson et al. (2022)	To examine caring experiences of kinship carers and their engagement with kinship support services.	Mixed methods study of carers, nonstandardized survey (*n* *=* 519) and interviews (*n* *=* 9).	Carer stress mostly associated with financial hardship, followed by children’s behavior problems, issues with birth parents, poor service by child protection agencies, and lack of cultural sensitivity and safety. Many carers indicated caring as very challenging (29%); most advised that caring was pleasurable with some challenges (42%).
Octoman and McLean (2014)	To explore foster carer support needs for managing children’s behavior.	Quantitative study of carers (*n* *=* 187), nonstandardized survey.	Carer stress mostly associated with lack of information from child protection services about children’s behaviors prior to fostering, poor relationships with child protection professionals, and insufficient respite services.
Randle et al. (2018)	To explain foster carer experiences of agency support and satisfaction.	Quantitative of carers (*n* *=* 137), modified Content-Validity Approach.	Carer stress associated with lack of agency support, preplacement training, financial support, and a poor pairing of the carer and the child.
Zuchowski et al. (2019)	To examine caring experiences of grandparent carers.	Qualitative study of birth families, carers, and workers (*n* *=* 77), interviews and focus groups.	Grandparent carer stress associated with lack of formal support and pressure from child protection services regarding kinship care arrangements.
Australia—Studies of interventions including ProQOL or its related concepts			
Study	Aim	Methods	Relevant Results
Frederico et al. (2014)	To evaluate the Circle Program (CP) focused on upskilling foster carers caring for traumatized children.	Qualitative study of carers (*n* *=* 28), specialists (*n* *=* 9), workers (*n* *=* 28) and support staff (*n* *=* 2), focus groups.	Improvements to specialist foster carer well-being associated with feeling well-trained, having more information about the children, and feeing more valued, compared to generalist foster carers.
Frederico et al. (2017)	To evaluate the outcomes of the CP.	Mixed methods study of CP children (*n* *=* 182), CP foster carers (*n* *=* 42), and general foster carers *(n* *=* 186); general foster carers (*n* *=* 38), and professionals (*n* *=* 56); mixed focus groups (*n* *=* 76).	CP had perceived associations with carers feeling supported and valued, which was increased carer retention, and less placement breakdowns or unplanned exits. Having support, training, ongoing education, and flexible access to funds and services, improved carer well-being.
Krishnamoorthy et al. (2020)	To explain change in caring experiences of carers in the 8-week Circle of Security-Parent Program.	Quantitative pre/post-study of carers (*n* *=* 54), SDQ, PSI, and Parent–Child Relationship Inventory (PCRI).	Intervention effect on total PSI scales and subscales were significant post-intervention, showing association between the program and reduced scores, but no covariate associations between subscales. SDQ subscales in clinical range pre- and post-intervention, small nonsignificant decrease in all subscales, and no significant co-variates. No intervention effect on PCRI scores.
United Kingdom—Studies including ProQOL or its related concepts			
Study	Aim	Methods	Relevant results
Anthony et al. (2019)	To explain anxiety, depression, and stress of parents of children adopted via the foster care system.	Quantitative study of carers/parents (*n* *=* 96) at 5-, 21-, 36-, and 48 month post-adoption, Hospital Anxiety and Depression Scale, Parenting Sense of Competence Scale, SDQ.	Higher parental depression score positively associated with child internalizing scores. Lower parental competency level predicted higher anxiety scores. Pre- and post-anxiety and depression scores relatively stable over the 4 years studied.
United Kingdom—Studies including ProQOL or its related concepts			
Study	Aim	Methods	Relevant results
Bridger et al. (2019)	To explain primary stress incidence and psychological predictors of secondary stress among foster carers.	Mixed methods study of carers (n = 187), ProQOL, Trauma History Screen, Toronto Empathy Questionnaire, Connor–Davidson Resilience Scale, Professional Self-care Scale, Trauma- Informed Self-Care, free-text questions.	Carer compassion satisfaction, burnout and secondary traumatic stress were higher than the reference sample (previous research in 2010). Burnout and compassion satisfaction were predictors for secondary trauma. Empathy, resilience, and self-care predictors of compassion satisfaction and burnout. Spending time with immediate family and friends was associated with improved well-being. Therapeutic support and specific training were mentioned as unmet support needs.
Hannah and Woolgar (2018)	To explain risk factors associated with foster and kinship carers’ intention to continue caring.	Quantitative study of carers (n *=* 131), ProQOL, Secondary Trauma Stress Scale, Acceptance and Action Questionnaire, White Bear Suppression Inventory, additional scaled questions.	Carers experienced compassion satisfaction (18%), burnout (18%), and high secondary traumatic stress (30%). Compassion satisfaction negatively associated with secondary trauma and burnout. High intention to continue caring associated with low burnout and secondary traumatic stress. High compassion satisfaction, low burnout, and secondary traumatic stress associated with job satisfaction. Psychological rigidity, thought suppression, and subsequent child trauma increased carer burnout.
Holt and Birchall (2022)	To explore experiences of grandparent kinship carers exposed to violence from their grandchild.	Qualitative study of carers (*n* *=* 27) and professionals (*n* *=* 9), interviews.	Secondary traumatic stress associated with the complex demands and emotional issues of the children. Additional stress associated with conflict with biological parents. Care stress compounded by their own traumas, for example, domestic violence, mental illness, addictions, and deaths.
Sloan Donachy (2017)	To explore associations between foster carer emotions and caring for a traumatized child.	Qualitative study of carers (*n* *=* 6), children’s social workers (*n* *=* 6), agency supervisors (*n* *=* 6), interviews.	Carer stress associated with time demands and interruption to daily life, worry, stress, and no relief, reporting they were hyper-vigilant/on edge, and experienced confusion, bewilderment, ambivalence, shocked, helpless, embarrassed, and discomfort.
United Kingdom—Studies of interventions including ProQOL or its related concepts			
Study	Aim	Methods	Relevant results
Begum et al. (2020)	To evaluate outcomes of an Attachment-Centered Parenting program for foster carers.	Mixed methods, carers (*n* *=* 10) in a 6-half-day weekly program, Carer Questionnaire, Parenting Sense of Competence, free-text questions.	Carer stress and anxiety levels associated with children’s behavior diminished post-intervention, linked to increased ability to practice therapeutic parenting, increased child responsiveness, confidence to manage children’s needs, and capacity to relate to the children.
Hutchings and Bywater (2013)	To explore challenges of caring from the perspective of leaders of the Incredible Years (IY) parenting program.	Qualitative study of program leaders (*n* *=* 5), reflective discussion about program participation.	Carer stress and mental health associated with children’s behavioral issues. The IY program resulted in improvements to children’s behaviors and reduced depression among carers.
Midgley et al. (2019)	To examine outcomes for foster carers Reflective Fostering (RF) Program group-based parenting training pilot.	Mixed methods, carers (*n* *=* 28), PSI, Parental Reflective Functioning Questionnaire (PRFQ), Brief Parental Self-Efficacy Scale, Goal-Based Outcome Measure (GBO), SDQ, BAC-C, Emotion Regulation Checklist (ERC), focus groups.	Statistically significant associations between carer stress, achievement of self-defined goals, child-focused emotional lability, and overall strengths and difficulties. Changes to reflective functioning of carers not significant, although improvements to reflective capacity was stated as meaningful by some carers at program completion.
Midgley et al. (2021)	To explain outcomes of an adaptation of the RF Program co-delivered by a social worker and experienced carer.	Mixed methods, carers (*n* *=* 38), PSI, PRFQ, GBO, SDQ, ERC, focus groups.	Statistically significant reductions to carers stress, carer-defined problems, and carer-reported measures of child difficulties shown at program completion. Carers advised co-delivery by a social worker and foster carer felt relevant to them and may have increased program efficacy.
Ranzato et al. (2021)	To explain the goals of foster carers in the RF Program Pilot.	Qualitative secondary analysis of carer (n *=* 26) data, GBO data after first program session.	Carers gained reflective capacity aimed at improving their own well-being, ability to manage their stress, to improve self-care, and communication and understanding of the children, emotions, and challenging behaviors. Engaging with other carers improved well-being.
Roberts et al. (2016)	To evaluate outcomes for foster and kinship carers, KEEPing Foster and Kinship Carers Trained and Supported program.	Quantitative, carers (*n* *=* 892), pre/post, 6-month and 12-month, Parent Daily Report (PDR), SDQ, Parenting Scale, and outcome data for 572 children.	Reduced carer stress post-intervention associated with positive changes in parenting, discipline, and improvement to children’s problem behaviors. Six- and 12-month post-intervention follow-up showed that significant improvements to behavioral difficulties, foster carer stress, and parenting discipline had been sustained.
United States—Studies including ProQOL or its related concepts			
Study	Design	Methods	Relevant results
E. R.Barnett et al. (2017)	To explore foster carers and adoptive parents’ perspectives on needs and services.	Mixed methods, carers, and parents via nonstandardized scaled survey (*n* *=* 512) and focus group (*n* *=* *27*).	Carer stress associated with lack of respect, poor communications, lack of preparation, lack of resources, and unrealistic expectations by child protection workers, welfare agencies and courts, and poor understanding by professionals on the needs of carers.
Barrett et al. (2021)	To explore lived experiences of parents adopting children from the foster care system.	Mixed methods, foster and adoptive families (*n* *=* 25), SDQ, PSI, focus groups (*n* *=* 59).	Carer stress associated with children’s behavioral difficulties, noted as 85% greater than children in general population. Stressors included insufficient support, resources, training, and communication, and being unfairly judged by family and friends.
Bundy-Fazioli et al. (2013)	To explore grandparents’ experiences of foster caring.	Qualitative study of grandparents (*n* *=* 15), survey, interviews, and focus group.	Carer stress and challenges were associated with responding to their grandchildren’s trauma and resilience, and setting limits with the children’s parents.
Cooley et al. (2015)	To explain impact of child disruptive behaviors on carer satisfaction.	Quantitative, carers (*n* *=* 155), Protective Factors Survey, Foster Parent Satisfaction Survey, Eyberg Child Behavior Inventory (ECBI), intention scaled question.	Carer stress reduced when tangible resources were available. Carer resilience increased when fewer children’s problem behaviors were reported. Resilience in itself was not significant in buffering the effects challenging children’s behaviors.
Denby et al. (2014)	To compare gender-based motivation as kinship carers, capacity, stress/strain, and family support.	Quantitative study, men carers (*n* *=* 70), women carers (*n* *=* 646), nonstandardized codesigned Kinship in Nevada (KIN) tool with 11 subscales.	Male carers showed high level of capacity/caring ability and low stress/strain compared to women. Capacity, stress and strain, and perceptions of child well-being, were associated with sociodemographic variables, not gender.
Fawley-King et al. (2020)	To compare internalized and externalized strain of biological, foster, and adoptive parents of youth receiving mental health services.	Quantitative, biological (*n* *=* 1,356), foster (*n* *=* 91), and adoptive (*n* *=* 173) parents, Caregiver Strain Questionnaire, Youth Services Survey for Families.	Carer stress had associations with the children’s mental health problems. Differences in externalized strain across the three carer types was not significant. Higher levels of social support were associated with less internalized and externalized strain in the three groups. Biological caregivers had significantly higher levels of internalized strain than foster or adoptive carers.
Gleeson et al. (2016)	To explain parenting stress among kinship carers associated with family competence, support, and resources.	Mixed methods, informal kinship carers (*n* *=* 207), surveyed at onset, 6, 12, and 18 months, PSI.	Stress was associated with levels of social support, family competence, and health of family members. Having family resources mediated the effects of low levels of family support and family competence on parenting stress.
Goemans et al. (2017)	To explain foster carer stress on children internalized and externalized behaviors and vice versa.	Quantitative, carer-children dyads (*n* *=* 237), SDQ, Nijmeegse Ouderlijke Stress Index Verkort.	Unidirectional prospective pathways showed that carer stress was associated with children’s internalizing and externalizing behaviors. There were no prospective pathways from parent stress to internalizing and externalizing behaviors.
Julien-Chinn et al. (2017)	To explain risks, strengths, relationships, and protective factors associated with foster family functioning.	Quantitative, carers (*n* *=* 681), Family Inventory of Life Events and Changes, Family Resilience and Strengths Scale, McMaster Family Assessment Device.	Compounding or elevated levels of risk of stress in carers were associated with poorer levels of family functioning. As family strength increased, so did the levels of healthy family functioning. Healthy family functioning improved well-being of foster children and associated well-being of carers.
United States—Studies including ProQOL or its related concepts			
Study	Design	Methods	Relevant results
Leathers et al. (2019)	To explain associations between parenting difficulties among foster carers of children with multiple placements.	Quantitative study of foster carers (*n* *=* 139), Ohio Youth Problem, Function and Satisfaction Scales, Support Functions Scale, additional scaled questions.	Children’s behavior, risk to others, low support, and stress were significant placement disruption predictors. Carer stress was associated with placement disruption outcomes. Higher disruption risk was observed among African American carers.
Lin (2018)	To explain associations between kinship carer stress and children’s engagement with mental health services.	Quantitative, carers (n = 1,623) data from 1999/2002 National Survey of America’s Families, Child Behavioral and Emotional Problem Scale, Parent Aggravation Scale, additional scaled questions.	Carer stress was linked to children’s behavioral problems. Social engagement moderated carer stress, that is, for carers of children aged 6–11, weekly engagement in volunteering buffered carer stress. Carer stress was significantly less among informal kinship families of children aged 17–12, compared to formal care.
Lopez et al. (2022)	To explain associations between carer social relations and stress.	Quantitative, carers (*n* *=* 65), Alabama Parenting Questionnaire, PSS.	Lower carer stress was associated with greater adult engagement with the children. High levels of stress was associated with higher acceptance of physical punishment and inconsistency in disciplining.
Miller and Grise-Owens (2021)	To measure COVID-19 impact on self-care of foster carers.	Quantitative pre/post survey of foster carers (*n* *=* 1,229), Self-Care Practice Scale.	Significant decline in self-care was associated with becoming a foster carer, with decline in self-care associated with poorer mental health. Financial stability was positively associated with good self-care. Married carers reported better mental health.
Nadeem et al. (2017)	To explain stress associated with children internalizing and externalizing problems.	Quantitative, children (*n* *=* 82) and their families, at 2 months, 12 months, and 5 years post-placement, Child Behavior Checklists (CBCL), PSI.	Carer stress was greater during children’s first year of placement. Early in placement, children >4-years of age had higher externalizing and internalizing problems than younger children. Differences were not significant 5-years after initial placement, length of placement associated with carer stress level.
Petrenko et al. (2019)	To explain stress looking after children with fetal alcohol spectrum disorders.	Qualitative study of carers (*n* *=* 24), interviews, and focus groups.	Carer stressed about the children’s disabilities, their social skills, system barriers, safety, development, and future life chances. Carer stress also associated with absence of social supports, family and marital issues, and social isolation.
Richardson and Futris (2019)	To explain carer couples’ stress, marital quality, and co-parenting relationship.	Quantitative, couples (*n* *=* 96), PSS, Quality of Marriage Index, Casey Foster Applicant Inventory-Applicant Co-Parenting Scale, and a Co-parenting Questionnaire.	Carer stress associated with perception of co-caring and its impact on and marital quality. Men’s marital quality mediated both men’s and women’s perceptions of associations between relationship quality. Marital relationship quality also mediated his perception of co-parenting quality, therefore his stress.
Schneiderman et al. (2012)	To explore carer help seeking, access, and use of healthcare for children.	Qualitative study, carers (*n* *=* 25), interviews.	Carer stress was associated with access to and satisfaction with services. Carers with financial stress had difficulty accessing primary health/Medicaid, and difficulty securing specialist health services.
Sharda et al. (2019)	To explain stress and social support associations among kinship carers.	Quantitative, kinship carers (*n* *=* 152), PSS, Multidimensional Scale of Perceived Social Support, Assessment of QoL.	Carer stress had negative association with well-being. Social support had positive association with well-being. Showed that social support did not moderate relationships between stress experienced by carers and their well-being.
Sharda (2022)	To explain links between carer stress, well-being, and social support.	Quantitative study of foster carers (*n* *=* 139), PSS, Mental Health Continuum, Social Provisions Scale.	Carer stress had negative association with well-being. Social support had a moderating effect in this interaction. Carers who reported high levels of social support were less adversely affected by parental stress than those who reported low levels of social support.
Shklarski (2019)	To explore carer stress experience and coping to resolve it.	Qualitative study of women carers (*n* *=* 11), interviews.	Key challenges associated with carer stress and coping were levels of attachment with the children, carer role confusion, and issues with respite services and with children’s biological families.
Sprang et al. (2015)	To explain child trauma exposure, grandparent stress, and mediating effect of child–grandparent conflict.	Quantitative study of carers (*n* *=* 251), PSS, SF-36-Item Health Survey, Child–Parent Relationship Scale, additional scaled questions.	Carer capacity, well-being and stress was indirectly associated with children’s trauma. Quantity of trauma exposures had associations with quantity of carer–child conflicts, but no association between with stress experienced by carers. High levels of conflict were linked to carer’s lower levels of emotional well-being.
White et al. (2021)	To explain carer or adoptive parent well-being, caring variables, and carer commitment.	Quantitative, carers (*n* *=* 937), Behavior Problem Index (BPI), Protective Factors Survey, Caregiver Strain Questionnaire-Adoption/Guardianship Form, additional scaled questions.	Carer stress associated with caring strain, child behavior difficulties, and lack of carer access to support services. Commitment, family nurturing, a kinship relationship, younger child age, and attachment were each associated with fewer child behavior problems and reduced caring strain.
Whitt-Woosley et al. (2020)	To explain factors associated with secondary traumatic stress of foster carers.	Quantitative study of foster carers (*n* *=* 1,161), Secondary Traumatic Stress Scale, *ProQOL.*	Most carers reported secondary traumatic stress in association with two forms of child trauma, with 77.8% of carers feeling some form of distress, thoughts, or adverse feelings. About 28.5% of carers describing these feelings as moderate to extreme, and 32.7% of them indicating that the stress impacted relational functioning and work functioning.
Woods (2021)	To explore experiences of African American grandmother carers with chronic health issues.	Qualitative, grandmothers (*n* *=* 8), interviews.	Carer stress associated with their own health, financial difficulties, limited support, transportation difficulties, and disruption to social activities.
Xu et al. (2022)	To explain grandparent material hardship, stress, psycho and social support, and resilience during COVID-19.	Quantitative, carers (*n* *=* 362), Mental Health Inventory-5, Brief Resilience Scale, Duke-University of North Carolina Functional Social Support Questionnaire (SSQ).	Carer stress mostly associated with material hardship; however, less chance of stress was associated with good relationships, social support, and resilience. Kinship status moderated associations between stress and relationships, and between social support and resilience.
United States—Studies of interventions including ProQOL or its related concepts			
Study	Aim	Methods	Relevant results
Adkins et al. (2018)	To explain intervention effect of Family Minds psychoeducational mentalizing program.	Random contraol trial (RCT), carers (*n* *=* 102, control group *n* *=* 48), PRFQ, 5-min Speech Sample coded using the Reflective Functioning scale (RF-FMSS), PSI.	Carer stress decreased post-intervention compared to control group. Increased understanding of children’s behaviors and reflective functioning had associations with lower stress.
Adkins et al. (2021)	To explain longitudinally the intervention effect of Family Minds psychoeducational mentalizing program.	RCT, carers *n* *=* 102, control group *n* *=* 48) at baseline, 6 weeks and 6 months follow-up, PRFQ, RF-FMSS, and PSI.	Carer stress, after 6 weeks of intervention, was lower in comparison to the control group. There were no significant differences between carer type, all had reduced stress associated with improved reflective functioning.
Bammens et al. (2015)	To explain intervention effect of Family Minds psychoeducational mentalizing program.	RCT, carers *n* *=* 17, control group *n* *=* 13, 5-min Speech Sample coded using Fonagy et al. (1998) reflective functioning coding manual.	Carer reflective functioning and understanding of their own emotions increased among intervention group participants, compared to the control group, potentially leading to better self- management of carer stress.
E. R. Barnett et al. (2019)	To examine effect of trauma-informed intervention on children behavior, carer and parent satisfaction and commitment.	Quantitative, carer and adoptive parents via nonstandardized survey (*n* *=* 512) and four focus groups (*n* *=* 27).	Trauma-informed services showed moderating effects on the associations between children’s behavioral problems and parent satisfaction in carers (but not in adoptive parents). As trauma-informed services increased, associations between child behavior and parent satisfaction were less strong, suggesting a buffering effect.
United States—Studies of interventions including ProQOL or its related concepts			
Study	Aim	Methods	Relevant results
Chakawa et al. (2020)	To explore tailoring and implementation of Parent–Child Interaction Therapy (PCIT) with an adoptive foster family.	Clinical case study of one multi-racial family with a child exhibiting behavior problems.	Carers less likely to become withdrawn, unresponsive to children’s needs, or placement breakdown, following PCIT. Carer-reported child behavior problems and increase in effective use of behavior management strategies following PCIT were clinically significant.
Feldman (2017)	To explain intervention effect of family group decision making (FGDM) conference on kinship carers and their families.	Quantitative pre/post study of carers, PSI, SSQ, Family Needs Scale, daily activity logs, scale items on safety and permanency; at 3-month follow-up, Client Satisfaction Questionnaire.	Phase one recruitment, Phase 2 case management only, Phase 3 case management/FGDM conference. Gain scores on measures of parenting stress, social support, family needs and child well-being were higher for Phase 3 families compared to Phase 2. Phase 3 families had higher CSQ-8 scores (not significant) at follow-up.
Furlong et al. (2021)	To assess the utility and perceived effectiveness of an 18-week IY parenting program.	Mixed methods, carers (n = 46), SDQ, PSI, Being a Parent scale, Warwick-Edinburgh Mental Well-being Scale. Interviews/focus groups with half the sample (n = 23).	Carer had reduced parenting stress associated with improved parenting competency and parent–child relationships. Statistically significant improvements in children’s social, emotional, and behavioral difficulties, as reported by foster and biological carers, likely associated with changes in parenting and stress.
Greeno et al. (2015)	To explain child behavior, parenting style, and carer stress in the KEEPing foster and kinship carers trained and supported program (KEEP).	Quasi-experimental pre/post study of carers (*n* *=* 65, comparison *n* *=* 48), CBCL, PDR, PSI, free-text questions on discipline type/frequency.	Carer PDR scores were lower, parents reported reduced CBCL severity levels in children, and significantly fewer child behavior problems on completing KEEP, compared to the control.
Maaskant et al. (2017)	To explain effectiveness of Parent Management Training Oregon (PMTO) for foster families with children who have severe externalizing behaviors.	RCT (Intervention carers, *n* *=* 46, control group *n* *=* 40), PSI, Parenting Behavior Questionnaire (PBQ), CBCL, ratings on experienced change.	Carers in the intervention group showed small to medium effect size reductions to stress, reduced child-related parenting stress, and reduced parent-related stress, compared to the control. Small effect size in PMTO helping foster mothers to maintain parental warmth. Child behavior problems reduced in both groups. Multilevel analyses showed no significant results.
Maaskant et al. (2016)	To explain sustained effectiveness of PMTO measured at 4 months follow-up.	RCT (Intervention carers, *n* *=* 46, control group *n* *=* 40), PSI, PBQ, CBCL, Parent Motivation Inventory, Working Alliance Inventory.	Multilevel analyses showed that post-intervention effect on parenting stress identified by Maaskant et al. (2017) had disappeared at 4-month follow-up. Likewise, PMTO had no significant or indirect effects at follow-up on parenting behavior and child behavior problems.
Mersky et al. (2015)	To explain intervention effectiveness of PCIT on carers’ attitudes toward children with severe behavior problems, and carer stress.	Waitlist control trial of carer-child dyads (*n* *=* 129), ECBI, PSI, Dyadic Parent–Child Interaction Coding System.	Significant decrease in self-reported parenting stress in the brief and the extended PCIT. Mixed effects generalized linear models showed PCIT to contribute significant improvements to positive and negative parenting indicators. The brief PCIT showed higher levels of effectiveness than the extended PCIT intervention.
Monahan et al. (2013)	To explore stress, burden, health, and service/support group use of grandparent and other kinship carers in the KinNET program.	Quantitative study of KinNET carers (*n* *=* 102), nonstandardized survey on participation, burden of caregiving, carer health status, perceptions of child safety and permanency planning.	Carer stress was associated with perceptions of caregiving burden as opposed to caregiving itself. Stress undermined carer health, and poor carer health was associated with increased stress, which likewise increased carer burden. Stress had not significant association with hours of care provided. Involvement in support groups was associated with high satisfaction.
Price et al. (2014)	To explain effectiveness of the KEEP program to reduce stress of carers of children with behavior problems.	Quasi-experimental pre/post-study of carers (*n* *=* 164, control *n* *=* 171), CBCL, PDR.	Reduction to carer stress in post-intervention results were significant. Hierarchical linear modeling showed the KEEP intervention was effective in reducing number of children engaged in rule-breaking, and in reducing internalizing problems. Children’s behavior change associated lower carer stress.
Richardson, Pettit, et al. (2021)	To examine the trait mindfulness of heterosexual foster carer couples in a relationship education program.	Quantitative, carer (*n* *=* 235) data from a larger study, Parenting Distress Scale, scaled items on emotions, actions, self-care.	Compared couple samples based on trait mindfulness: men low/women high, men low/women high, man high/women low, men high/women high. The men high/women low subsample showed significantly higher stress compared to three other groups. Men had less stress when women had high levels of mindfulness.
Strozier (2012)	To explain the effectiveness of the kinship carer group on social supports.	Quasi-experimental, carers (intervention *n* *=* 40, control *n* *=* 20), Dunst Family Support Scale.	Carer well-being was higher among those attending support groups and who experienced increases in social support for the duration. Kinship carers attending the support groups experienced increased formal social support than informal social support.
Richardson, Mallette, et al. (2021)	To explain effectiveness of a Couple Relationship Education program, and associated change in relationship quality.	Quantitative, carers (*n* *=* 266), PSS, Casey Foster Applicant Inventory-Applicant Co-Parenting Scale, Co-parenting Questionnaire.	The intervention moderated positive change in stress for the men, not the women, albeit both experienced perceptions of positive change in their co-parenting support.

### Carer ProQOL, Aspects, and/or Related Concepts

**Figure 2.**
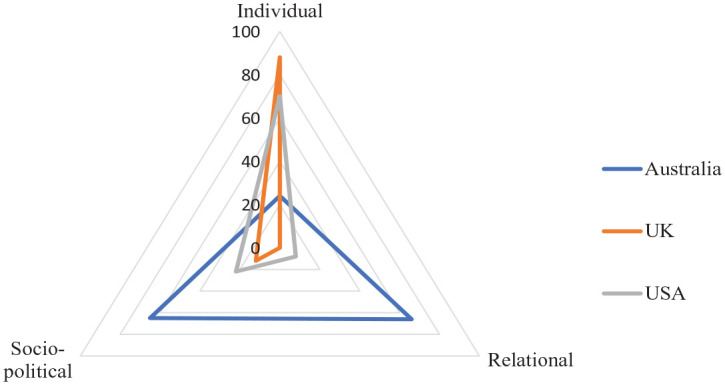
Percentages of studies at the individual, relational, and sociopolitical levels.

Studies across the three countries differed in terms of their focus on individual, relational, or sociopolitical aspects associated with ProQOL and/or related outcomes for carers (Figure 2). More than 85% of studies from the United Kingdom (*n* *=* 10) and 70% from the United States (*n* *=* 29) had their interest in individual factors associated with carer ProQOL or its related concepts, compared to Australian research that focused more heavily on relational or sociopolitical factors.

### Studies Focused on Individual Factors

Several studies focused on individual factors and reported the impact of carer’s interpersonal relationships, roles, and activities on their career well-being. Australian studies in particular highlighted the difficulty carers face in fulfilling their responsibilities and meeting high expectations. For instance, Breman et al. (2018) found that the role demands associated with children’s challenging behaviors were the primary cause of stress and burnout among carers. Some studies have indicated a link between children’s emotional and behavioral issues and the difficulties and stress carers face in meeting their needs (Fergeus et al., 2019a, 2019b; Harnett et al., 2014; McKeough et al., 2017). Others reported that carers often find themselves caught in conflicts among fostered children or between fostered children and their own other close family members, requiring carers to intervene which adds to their pressures (Harnett et al., 2014; Kiraly et al., 2020; McKeough et al., 2017). Stress is amplified when carers lack adequate time for themselves (Fergeus et al., 2019a).

Studies in the United Kingdom have likewise demonstrated a correlation between behavioral problems in children and poor psychological well-being of carers (Anthony et al., 2019; Holt & Birchall, 2022; Morgan & Baron, 2011). In relation to ProQOL-related concepts, two studies found that caring for traumatized children frequently leads to secondary traumatic stress for the carers (Bridger et al., 2019; Hannah & Woolgar, 2018). Sloan Donachy (2017) reported that caring for traumatized children can lead to conflicting emotions, being constantly on edge, and a loss of positive self. It was also revealed that burnout, compassion satisfaction, and primary trauma had direct effect and self-care had indirect effect on carers’ secondary traumatic stress (Bridger et al., 2019), and that psychological inflexibility and thought suppression were positively associated with both carers’ secondary traumatic stress and burnout, which were negatively associated with carers’ intent to continue fostering (Hannah & Woolgar, 2018). Anthony et al.’s (2019) longitudinal study indicated that carers’ sense of competence was negatively associated with initial anxiety and depression and positively associated with a steeper decline of depression over 4 years post-placement. Other factors that may contribute to poor ProQOL include a lack of support from family and friends and conflict with children’s birth families (Bridger et al., 2019; Holt & Birchall, 2022). Consistent with Australian studies, the high demands placed on UK carers to manage children with complex problems also adds to their stress (Holt & Birchall, 2022; Sloan Donachy, 2017).

Many US studies focused on internalizing and externalizing behaviors of children as the major source of stress for carers (Barrett et al., 2021; Bundy-Fazioli et al., 2013; Cooley et al., 2015; Fawley-King et al., 2020; Goemans et al., 2017; Leathers et al., 2019; Lin, 2018; Nadeem et al., 2017; White et al., 2021). Two studies linked high family dysfunction and low family competence to carer strain (Gleeson et al., 2016; Julien-Chinn et al., 2017). In addition, relationship have been found between judgmental attitudes of families and friends and high carer stress ( E.R.Barnett et al., 2017; K. C. Barnett et al., 2021). On the other hand, support from family and friends was found to be associated with low stress levels in multiple studies (Fawley-King et al., 2020; Gleeson et al., 2016; Leathers et al., 2019; Lin, 2018; Sharda, 2022; Sharda et al., 2019; Woods, 2021). Several studies reported that factors such as ethnicity, age, marital status, financial status, and mental health status predict carer contentment (Denby et al., 2014; Leathers et al., 2019; Miller & Grise-Owens, 2021; Richardson & Futris, 2019). Lack of resilience was also identified as a predictor of high stress in three studies (Bundy-Fazioli et al., 2013; Cooley et al., 2015; Xu et al., 2022).

Two studies from the United States showed associations between carer stress and maladaptive parenting behaviors such as poor monitoring, inconsistent discipline, and low attachment or engagement with children (Lopez et al., 2022; Shklarski, 2019). The stress levels experienced by carers was shown in other studies to be influenced by the age of the children in care and the placement phase, with younger children and initial placement being predictors of higher stress level (Nadeem et al., 2017; White et al., 2021), as were children’s trauma and their conflicts with adult children in the household (Bundy-Fazioli et al., 2013). It was also reported that couple parenting stress in foster families was related to the perceptions of both parent and their partners regarding their marriage and the co-parenting relationship (Richardson & Futris, 2019). In addition, research revealed a variety of factors associated with caring stress including advanced age, poor overall health, and limited social life among grandparent carers (Sprang et al., 2015; Woods, 2021).

### Studies Focused on the Relational Level

Regarding the factors at relational level, many studies examined relations between carers and entities having control over their care work, with most informed by research in Australia. Two Australian studies showed that poor communication by child welfare workers was associated with carer stress and feelings of incompetence (Kiraly et al., 2015; Octoman & McLean, 2014). The actual interference by child welfare workers, involving critique of parenting, was also reported to contribute to feelings of incompetence among carers (Blythe et al., 2013). Furthermore, the authoritative and reductionist attitudes of workers were argued by Fergeus et al. (2019b) to create conflicts for carers when directives were perceived not in the child’s best interests. Three studies expanded on the notion of poor communication in contexts where cultural sensitivity was imperative, observing that a lack of sensitivity by child protection workers created additional tensions for carers (Kiraly et al., 2015; McPherson et al., 2022; Randle et al., 2018). Likewise, the UK study by Bridger et al. (2019) showed a perceived a lack of practical and therapeutic support from their professional environment in addition to that received from family and friends.

Studies from the United States have found that poor communication between child protection workers and carers led to frustration for carers, as it inhibited children’s access to necessary services ( E.R.Barnett et al., 2017; K. C. Barnett et al., 2021; Schneiderman et al., 2012). Research also showed that unrealistic expectations, demands, and lack of respect communicated by court authorities, child welfare agencies, and other professionals toward carers contributed to their stress ( E.R.Barnett et al., 2017). These relational factors were linked to poor carer–child pairing in the Australian study by Randle et al. (2018), resulting in higher rates of placement breakdowns and stress for both children and carers.

### Studies Centered at the Sociopolitical Level

The governance of social provision processes, including the foster and kinship care system, is managed at the sociopolitical level. While the foster system prioritizes the welfare of children over economic benefits, in practice, there is a struggle between authorities. Australia, the United Kingdom, and the United States have different approaches, with Australian research reporting more on the stressors at the sociopolitical level. There was an absence of UK studies addressing the factors associated with carer’ ProQOL at sociopolitical level.

Blythe et al. (2013) argued that caring for children was challenging and stressful when dealing with complex sociopolitical issues. They advised that child protection systems were bounded in policy red-tape informing practice that is not appreciative of the complexity involved in caring (Blythe et al., 2013). This finding was consistent with the range of Australian studies that demonstrated policy informing child protection practice characterized by abundant home visits, heavy scrutiny of carers, promises of supports not given, and insufficient financial resources resulting in inadequate care (Blythe et al., 2013; Harnett et al., 2014; Kiraly et al., 2020; McPherson et al., 2022; Randle et al., 2018). Two studies reported carer feelings of being dictated to, thereby diminishing their rights to make decisions related to the children in their care (Fergeus et al., 2019a; Octoman & McLean, 2014). Carers in these studies were denied access to historical information about the children, which could have better enabled their approach to child-centered care (Fergeus et al., 2019a; Octoman & McLean, 2014). Denied access to historical information was linked to difficulties, frustration, and burnout in trying to navigate services and supports with the children (Fernandes et al., 2021; Kiraly et al., 2015; McPherson et al., 2022). Australian research generally showed that carers wanted greater autonomy to do things that are in the best interests of foster children, along with more support, such as the support to promote mental health children in their care, the support from the government, and accessing respite (Fergeus et al., 2019b; Fernandes et al., 2021; Octoman & McLean, 2014; Randle et al., 2018; Zuchowski et al., 2019).

Several Australian studies (Harding et al., 2020; McKeough et al., 2017; Randle et al., 2018) also showed that informal carers, specifically grandparent carers, have a crucial role to play in supporting children’s lives, but they face heavy burden that can negatively impact their health and received little support. Blythe et al. (2012) noted that sociopolitical factors can lead to carers being treated like second-class parents. The UK study by Bridger et al. (2019) showed that the provision of a range of appropriate resources and supports could uplift carers’ political status and individual well-being, nonetheless these interact across level impacting the sense of well-being at the relational. level. A few US studies focused on sociopolitical factors associated with carer stress emphasized poor governance and inadequate access to primary and specialty services for children and young people in care ( E.R.Barnett et al., 2017; Schneiderman et al., 2012; White et al., 2021; Woods, 2021), resulting in an unstable support system for carers (Barrett et al., 2021). Although training was provided, a lack of post-training resources left carers feeling unsupported (Barrett et al., 2021). Carers reported a lack of systemic supports and resources as a source of stress (Cooley et al., 2015), including barriers in obtaining health insurance and transportation for the children (Schneiderman et al., 2012; Woods, 2021; Xu et al., 2022).

### Interventions Reporting Outcomes on ProQOL or Related Concepts

Improvement to ProQOL or its related concepts associated with carer interventions was described in 27 studies across the three countries. We explored and reported herewith the impact and significance of interventions reported in relation to ProQOL or its related concepts among foster and kinship carers. Meta-analysis of the effectiveness of these interventions in terms of reducing secondary stress, burnout, and compassion satisfaction falls outside of the scope of our review. We note, however, that there are gaps between the caring stress-related problems experienced by carers and the interventions available to them.

Our search located three studies from Australia. One, known as the CP, is a therapeutic foster care program that reportedly has positive impact on the outcomes of children in care who have experienced trauma. Specific to the carers, Frederico et al. (2014) evaluated the CP, proposing that it was therapeutic for reason of offering carers a voice. Key outcomes were that the carers believed they were well-trained, had personal knowledge of the children, and felt personally valued. In a later mixed methods analysis, Frederico et al. (2017) demonstrated associations between therapeutic foster care training and carer retention, fewer foster child placement breakdowns, and unexpected exits. They reported that carers who participated in the program liked the assistance, training, continuous education, and flexible financial access. The third Australian intervention study, the Circle of Security-Parent Program, was evaluated by Krishnamoorthy et al. (2020). While program outcomes showed little impact on parent–child relationships, positive impact on overall carers stress was substantial.

Among the six UK studies reporting interventions, there were four intervention types: attachment-centered parenting (Begum et al., 2020), IY Parenting Program (Hutchings & Bywater, 2013), Reflective Fostering Program (Midgley et al., 2019, 2021; Ranzato et al., 2021), and KEEP—Keeping foster and kinship carers trained and supported program (Roberts et al., 2016). All were designed to reduce the negative effect of children’s challenging behaviors on the psychological well-being of carers. The attachment-centered parenting program showed increased carer confidence to manage children’s needs and implement therapeutic parenting as outcomes (Begum et al., 2020). Program outcomes of IY was significant reduction in child problem behaviors in association with lower levels of depression among the carers (Hutchings & Bywater, 2013). Three evaluations of the Reflective Fostering Program, involving only carers, indicated improved levels of stress associated with applying training with children in their care who had emotional and behavioral problems (Midgley et al., 2019, 2021; Ranzato et al., 2021). Roberts et al. (2016) examined the outcomes of KEEP programs, which demonstrated significantly lower carer stress upon program completion.

Program interventions with carers in the United States also showed improvements post-intervention (i.e., Chakawa et al., 2020; Mersky et al., 2015). The Family Minds program that aimed to improve reflective functioning of carers indicated decreased stress levels at program completion (Adkins et al., 2018, 2021). Feldman (2017) evaluated the Family Group Decision Making program, showing reduced parenting stress and improvements in social support, family needs, and child well-being. Furlong et al. (2021), who evaluated IY, reported improvements to children’s social, emotional, and behavioral difficulties, parenting competency, and carer stress. Price et al. (2014), likewise, found positive correlation between children’s problem behaviors and carer stress associated with engagement in the KEEP training program.

Many interventions focused on training carers to manage children’s adverse behaviors, involving pre- and post-program measures via RCT or quasi-experimental methods, only one study sought understanding of longer-term outcomes. This was an RCT of the Parent Management Training Oregon involving 9-months of intensive intervention in the United States with carers and children (Maaskant et al., 2017). Measures upon program completion reported that child-related parenting stress and parent-related stress had declined (Maaskant et al., 2017), but these intervention effects had worn off by 4-month follow-up (Maaskant et al., 2016). Several other studies reported mixed results, including Monahan et al. (2013) study of grandparent and kinship carers showing high levels of satisfaction with program interventions, but the impact of the intervention on reducing caregiving stress and burden was inconclusive.

A few interventions directly with carers focused on self-management of psychological well-being. Richardson, Pettit et al. (2021), for example, examined the effect of mindfulness training in relation to parenting stress and adult relationship stress and found that mindfulness contributed to foster couple caregivers’ relationship quality and individual well-being. Richardson, Mallette et al. (2021), however, reported gender differences in a co-parenting intervention. It was indicated that while both men and women experienced perceptions of positive change in support from their co-parent, there were higher levels of moderated positive change in parenting stress among men but not women.

According to Strozier (2012), attending carer peer groups led to significant increases in social support for carers. Interestingly, out of 16 US intervention studies reviewed, the majority focused on individual-level interventions, with only one by E.R.Barnett et al. (2019) examining the impact of trauma-informed mental health services on the relationships between carers and children. The findings of this study suggest that such services can play an important role in moderating these relationships.

## Discussion

This review revealed that a variety of factors at the individual, relational, and sociopolitical levels were associated with ProQOL or its related concepts among foster and kinship carers as reported in research undertaken in Australia, United Kingdom, and United States. There were also many different interventions associated with the improvement of ProQOL and its related concepts for foster and kinship carers as reported in research undertaken in Australia, United Kingdom, and United States. However, findings showed that insufficient attention has been paid to the determinants of ProQOL or its related concepts at the relational and sociopolitical levels. In the United Kingdom and United States, research have primarily located the source of carer stress at the individual level. In contrast, the Australian research acknowledged that carer ProQOL and its related concepts were more complex and paid attention to the determinants at sociopolitical level. The review also demonstrated that while supports and interventions were available, they were not necessarily supports and interventions that meet the needs of carers for their quality of life or well-being, nor was it known if interventions having some success at improving carer ProQOL and/or its incumbent variables sustained stress reduction beyond the interventions themselves. Only one post-intervention and longitudinal study demonstrated that intervention effect for carers was not sustained (Maaskant et al., 2016), indicating that some sort of ongoing intervention into carer stress, post-program completion, as well as more longitudinal search on such interventions are needed.

Our review highlights the importance of considering the ProQOL and its related concepts of carers, as the stress and trauma they experience when caring may affect the quality of care they provide. There are numerous stressors that caregivers face, such as their childhood trauma, attachment issues in their childhood, and their emotional instability and these affect their ability to provide care (Bryant et al., 2017; Rassart et al., 2022). Interestingly, caregivers who had experienced childhood trauma reported lower levels of child dissociation symptoms than their counterparts (Cohodes et al., 2016), indicating that lived experience may assist carers in their work and coping with associated stress. In light of these findings, effort to reduce stress, or enhance carer ProQOL, would benefit from understanding carers’ home contexts and their own histories of adversity.

We identified that children’s behavioral problems were the primary predictor of carers’ stress in studies from across the three countries. While many children in care may have delayed developmental progress associated with abuse and placement instability, parenting interventions with carers appropriately focus on safety, supports, and stability of the children (Jacobsen et al., 2020; Sattler et al., 2018; Tenenbaum et al., 2020; Wade et al., 2020). These studies focused discretely on improving parenting capacity as a mechanism to reduce stress, with limited acknowledgment of other factors that may also be contributing to stress in people’s lives.

Fostering is a professional or semi-professional role that has become more complex with legislation and regulatory reforms (Smyth & McHugh, 2006; Vanderfaeillie et al., 2016). This has led to increased demands on carers and the need for ongoing education, training, and support (De Wilde et al., 2019). Studies from the United Kingdom and the United States are more concentrated in training carers, and individual change related to managing dyadic adult–child relationship with emphasis on improving the caring capacity, carer experiences, and ProQOL. In contrast, Australian studies are predominantly focused on stressors at the sociopolitical level. This is due in part to significant historical contexts and ongoing intergenerational trauma experienced by survivors, their families, and the descendants of Australia’s Stolen Generations (Ireland, 2018) and in part the ongoing associations with overrepresentation of Aboriginal and Torres Strait Islander children experiencing statutory removal from their families (Australian Institute of Health and Welfare, 2020). These contexts might facilitate Australian researchers to focus more on the factors associated with foster and kinship carers’ stress and ProQOL at the sociopolitical level, rather than on the individual and relational levels.

Whether the research focuses on individual, relational, or sociopolitical change, it is important to recognize that caring is present and necessary in both private and public domains. Looking after children in foster or kinship care is not merely a matter of personal or family responsibility of couples or of women who take them on. Caring is an essential part of society that involves both individual and collective efforts in meeting the needs of those who require care (Fisher & Tronto, 1990; Hugman, 1991). UK and US studies with focus on carer stress largely focused on individual-level change, while sociopolitical and relational factors appear to be of less focus. The UK and US studies in our sample showed the contribution of interpersonal factors to carer stress that may lead to placement breakdown or carer attrition. Interactions with family, friends or the children’s biological families were also stated as impacting role satisfaction among carers (Miller et al., 2019).

Additionally, personal circumstances, children’s characteristics, an unsupportive family, and fear of negative judgment are more broadly noted in literature to discourage people from becoming carers (Delfabbro et al., 2008). To address these gaps, researchers propose socio-ecological and ecosocial frameworks that consider multiple levels of influence on carer health and well-being (Schölmerich & Kawachi, 2016). According to Wendt’s (2021) ecosocial framework, carers’ ProQOL or well-being is influenced not only by individual and environmental factors but also by the interactions between them. This means that research seeking understanding of, and interventions to support, carers must consider broader institutions and structures in which they are embedded, as well as their unique beliefs and practices. To create sustainable improvements in carer health, well-being or ProQOL, it’s necessary to target all of these factors simultaneously, rather than simply emphasizing individual responsibility to cope better with stress.

We note that care systems in the United States were set up in a way that left carers entirely reliant on government resources and supports, which were inadequate (Schneiderman et al., 2012). Research indicated that the COVID-19 pandemic had exposed and exacerbated existing problems (Miller & Grise-Owens, 2021). Care systems in United Kingdom and Australia are also heavily reliant on government resources. Albeit, more resources and supports seem critical to enhance foster care systems and help promote carers’ ProQOL.

## Limitations

This scoping review has a few limitations. First, the focus on ProQOL and the inclusion of stress and well-being as ProQOL subscale-related concepts might have resulted in underrepresentation of research which examined the factors associated with foster and kinship carers’ anxiety, post-traumatic stress disorders, and other well-being-related concepts in this review. Second, following Arksey and O’Malley (2005) scoping review methodology, the quality of the reviewed literature was not assessed in this research. Third, we included studies conducted in just three countries and excluded the literature in languages other than English. We also did not search the unpublished literature. Thus, there was potential for publication bias in this review. Fourth, the process of scoping the literature involved interpretation, as we did not have direct access to the source knowledge in the same way as the authors who produced the knowledge, there might be interpretation bias in this review. Notwithstanding the limitations, the findings provide a foundation for future research and policy interventions aimed at improving the ProQOL or well-being of foster and kinship carers. Our review also signals the need for further research on multiple levels of influence on the ProQOL and well-being of carers.

## Conclusion

This is the first scoping review to provide an overview of empirical research on the factors associated with ProQOL and its related concepts as well as the interventions to improve these for foster and kinship carers. We explored the literature in Australia, United Kingdom, and United States and revealed that carers faced stress at the individual, relational, and sociopolitical levels and that there were a variety of interventions associated with the improvement of carers’ ProQOL or well-being. The implications of the review for research, policy, and practice were summarized in Table 3. In conclusion, this study highlights the significant burden and stress experienced by carers and the potential strategies for policy and practice to alleviate these challenges. To develop a better understanding about the factors associated with foster and kinship carers’ ProQOL or well-being, it is necessary to target different factors at individual, relational, and sociopolitical levels, and the interactions among them simultaneously. To reduce carers’ burnout and stress and enhance their compassion satisfaction, efforts should also be made to develop relational-level and sociopolitical level interventions.

**Table 3.** Summary of Critical Findings and Implications.

Critical findings of this review
Studies indicated that building capacity of foster and kinship carers to undertake their work has potential to improve the environment of children and young people in care, mitigate carer attrition, and contribute to placement stability.
Many pre- and post-studies indicate improvements in carer professional quality of life (ProQOL) and its related concepts through training and support interventions However, limited evidence exists from this review on whether interventions have longer-term intervention effect. Only one study measured outcomes at 4-month follow-up showing interventions effect had disappeared. This calls to question the sustainability of interventions beyond post-measurement.
Studies reviewed did not report on interaction between individual, relational, and sociopolitical factors in relation to foster and kinship carer ProQOL or aspects of ProQOL, or related concepts. This has consequences for understanding the influence of confounding factors across system levels on outcomes reported.
Implications for policy, research, and practice
More research is warranted to examine the determinants of foster and kinship carer ProQOL or its related concepts at individual, relational, and sociopolitical levels as well as the interaction among them simultaneously. The multilevel burden of caring on carers highlights the importance of multileveled social interventions, particularly at the relational and sociopolitical levels.
Longitudinal evidence on what may improve the work and lives of foster and kinship carers over the longer-term is relatively limited. Post-intervention follow-up is needed for understanding sustained intervention effect, and to inform policy and best practice.
Evidence-based supports across system levels, for carers, is needed to reduce attrition, and increase stability for children and young people requiring care.
Supporting effective communication, information sharing, and coordination among carers, agencies, child welfare service providers, and statutory organizations is essential.

## Supplemental Material

sj-docx-1-tva-10.1177_15248380231213322 – Supplemental material for Professional Quality of Life of Foster and Kinship Carers in Australia, United Kingdom, and the United States: A Scoping Review Supplemental material, sj-docx-1-tva-10.1177_15248380231213322 for Professional Quality of Life of Foster and Kinship Carers in Australia, United Kingdom, and the United States: A Scoping Review by Helen McLaren, Emi Patmisari and Yunong Huang in Trauma, Violence, & Abuse
