# Supplementary material for: Professional Quality of Life of Foster and Kinship Carers in Australia, United Kingdom, and the United States: A Scoping Review
Source: Trauma Violence Abuse. 2023 Dec 1;25(3):2390–406. doi: 10.1177/15248380231213322 (PMC11155225; doi:10.1177/15248380231213322)
Supplement: sj-docx-1-tva-10.1177_15248380231213322 – Supplemental material for Professional Quality of Life of Foster and Kinship Carers in Australia, United Kingdom, and the United States: A Scoping Review [file sj-docx-1-tva-10.1177_15248380231213322.docx]

Supplementary 1. Search strategy for each database

| TITLE-ABS-KEY ("foster care" OR "foster children" OR "out-of-home care" OR "kinship care" OR "looked after children" OR "kinship fostering" AND carer OR caregiver OR parent OR grandparent OR custodian OR guardian AND "quality of life" OR "health-related quality of life" OR wellbeing OR well-being OR satisfaction OR fatigue OR "compassion fatigue" OR exhaustion OR "occupational stress" OR burnout OR burn-out OR stress OR trauma OR "secondary traumatic stress" OR "negative affect" OR "positive affect" AND intervention OR treatment OR management OR model OR policy OR practice OR guideline) AND PUBYEAR > 2011 | Scopus  (669 results) |
| --- | --- |
| ab("foster care" OR "foster children" OR "out-of-home care" OR "kinship care" OR "looked after children" OR "kinship fostering") AND ab(carer OR caregiver OR parent OR grandparent OR custodian OR guardian) AND ab("quality of life" OR "health-related quality of life" OR wellbeing OR well-being OR satisfaction OR fatigue OR "compassion fatigue" OR exhaustion OR "occupational stress" OR burnout OR burn-out OR stress OR trauma OR "secondary traumatic stress" OR "negative affect" OR "positive affect") AND ab(intervention OR treatment OR management OR model OR policy OR practice OR guideline) 2012-2022 | ProQuest  (564 results) |
| (“foster care” OR “foster children” OR “out-of-home care” OR “kinship care” OR “looked after children” OR “kinship fostering” ) AND ( carer OR caregiver OR parent OR grandparent OR custodian OR guardian ) AND ( “quality of life” OR “health-related quality of life” OR wellbeing OR well-being OR satisfaction OR fatigue OR “compassion fatigue” OR exhaustion OR “occupational stress” OR burnout OR burn-out OR stress OR trauma OR “secondary traumatic stress” OR “negative affect” OR “positive affect" ) AND ( intervention OR treatment OR management OR model OR policy OR practice 2012-2022 | CINAHL  (364 results) |
| ("foster care" or "out of home care" or "kinship care").mp. [mp=title, abstract, heading word, table of contents, key concepts, original title, tests & measures, mesh word] AND (carer or caregiver or parent or grandparent or custodian or guardian).mp. [mp=title, abstract, heading word, table of contents, key concepts, original title, tests & measures, mesh word] AND (intervention* or treatment* or management or model).mp. [mp=title, abstract, heading word, table of contents, key concepts, original title, tests & measures, mesh word] AND limit to peer reviewed journal english language and yr="2012 - 2022" | PsycINFO  (423 results) |
| "foster care" OR "foster children" OR "out-of-home care" OR "kinship care" OR "looked after children" OR "kinship fostering" (Title) AND carer OR caregiver OR parent OR grandparent OR custodian OR guardian (Topic) AND intervention OR treatment OR management OR model OR policy OR practice OR guideline (Topic) AND "quality of life" OR "health-related quality of life" OR wellbeing OR well-being OR satisfaction OR fatigue OR "compassion fatigue" OR exhaustion OR "occupational stress" OR burnout OR burn-out OR stress OR trauma OR "secondary traumatic stress" OR "negative affect" OR "positive affect" (Topic) 2012-2022 | Web of Science  (236 results) |
| ((("foster care"[Title/Abstract] OR "foster children"[Title/Abstract] OR "out-of-home care"[Title/Abstract] OR "kinship care"[Title/Abstract] OR "looked after children"[Title/Abstract] OR "kinship fostering"[Title/Abstract]) AND (carer OR caregiver OR parent OR grandparent OR custodian OR guardian)) AND ("quality of life" OR "health-related quality of life" OR wellbeing OR well-being OR health-related OR satisfaction OR fatigue OR "compassion fatigue" OR exhaustion OR "occupational stress" OR burnout OR burn-out OR stress OR trauma OR "secondary traumatic stress" OR "negative affect" OR "positive affect")) AND (intervention OR treatment OR management OR model OR policy OR practice OR guideline) 2021-2022 | PubMed  679 (results) |
| [[Abstract "foster care"] OR [Abstract "foster children"] OR [Abstract "out-of-home care"] OR [Abstract "kinship care"] OR [Abstract "looked after children"] OR [Abstract "kinship fostering"]] AND [[All carer] OR [All caregiver] OR [All parent] OR [All grandparent] OR [All custodian] OR [All guardian]] AND [[All "quality of life"] OR [All "health-related quality of life"] OR [All wellbeing] OR [All well-being] OR [All satisfaction] OR [All fatigue] OR [All "compassion fatigue"] OR [All exhaustion] OR [All "occupational stress"] OR [All burnout] OR [All burn-out] OR [All stress] OR [All trauma] OR [All "secondary traumatic stress"] OR [All "negative affect"] OR [All "positive affect"]] AND [[All intervention] OR [All treatment] OR [All management] OR [All model] OR [All policy] OR [All practice] OR [All guideline]] | SAGE Journals  (669 results) |
| ("foster care" OR "out of home care" OR "kinship care" OR "looked after") AND (carer OR caregiver OR parent) AND ("quality of life" OR wellbeing) AND (intervention OR treatment) | JSTOR  (340 results) |
